# Supplementary material for: Heat adaptation of phage T7 under an extended genetic code
Source: Virus Evol. 2021 Dec 1;7(2):veab100. doi: 10.1093/ve/veab100 (PMC8923235; doi:10.1093/ve/veab100)
Supplement: veab100_Supp [file veab100_supp.zip › Sequencing_Analysis.zip!/Sequencing_Analysis/A3/evidence/RA_17.html]

BRESEQ :: Evidence


| Read alignment evidence... | | | | | | | | | | | |
| --- | --- | --- | --- | --- | --- | --- | --- | --- | --- | --- | --- |
|  | seq id | position | | ref | new | freq | score (cons/poly) | reads | annotation | genes | product |
| \* | NC\_001604.1 | 167 | 0 | T | A | 48.9% | 41.8 / inf | 1414 | intergenic (–/–) | *–/–* | –/– |
| Reads supporting (aligned to +/- strand):  **ref** base T (413/306);  **new** base A (423/266);  **total** (837/577) | | | | | | | | | | | |
| Fisher's exact test for biased strand distribution *p*-value = 1.43e-01 | | | | | | | | | | | |
| Kolmogorov-Smirnov test that lower quality scores support variant *p*-value = 7.45e-01 | | | | | | | | | | | |

Only 100 of 1428 total aligned reads displayed.  

`‑‑‑‑‑‑‑‑‑TCTCACAGTGTACGGACCTAAAGTT‑CCCCCATAGGGGGTACCTAAAGCCCAGCCAATCACCTAAAGTCAACCTTCGGTTGACCTTGAGGGTTCCCTAAGGGTTGGGGATGACCCTTGGGTTTGTCTTTGGGTGTTACCTTGAGTGTCTCTCTGTGT‑C‑CCTATCTGTTACAGTCTCCTAAAGTATCCTCCTAAAGTCACCTCCTAACGTCCATCCTAAAGCC‑AACACCTAAAGCCTACACCTAAAGACCCATCAAGTCAACGCCTATCTTAAAGTTTAAACA‑‑‑‑‑‑‑‑‑‑‑‑‑TAAAGACCAGACC‑TAAAGACCAGACCTAAAGACACTACATAAAGACCAGACCTAAAGACGCCTTGTTGTTAGCCATAAAGTGATAACCTTTAATCATTGTCTTTATTAATACAACTCACTATAAGGAGAGACAACTTAAAGAGACTTAAAAGATTAATTTAAAATTTATCAAAAAGAGTATTGACTTAAAGTCTAACCTATAGGATACTTAC  >  NC_001604.1/1‑493`  

                                                                                                                                                                                  `|`                                                                                                                                                                                                                                                                                                                                                       
`gatgtgtagaagagagaggtACGGACCTAAAGTT‑CCCCCATAGGGGGTACCTAAAGCCCAGCCAATCACCTAAAGTCAACCTTCGGTTGACCTTGAGGGTTCCCTAAGGGTTGGGGATGACCCTTGGGTTTGTCTTTGGGTGTTACCTTGAGGGTCTCTCTGTGT‑C‑CC‑ATCTGTTACAGTCTCCTAAAGTATCCTCCTAAAGTCACCTCCTAACGTCCATCCTAAAGCC‑AACACCTAAAGCCTACACCTAAAGACCCATCAAGTCAACGCCTATCTTAAAGTTTAAACA‑‑‑‑‑‑‑‑‑‑‑‑‑taaagaccagac                                                                                                                                                                                                           <  1:264582/283‑1 (MQ=255)`  
 `gtgtataagagacaggtgtACGGACCTAAAGTT‑CCCCCATAGGGGGTACCTAAAGCCCAGCCAATCACCTAAAGTCAACCTTCGGTTGACCTTGAGGGTTCCCTAAGGGTTGGGGATGACCCTTGGTTTTGTCTTTGGGTGTTACCTTGAGTGTCTCTCTGTGT‑C‑CCTATCTGTAACAGTCTCCTAAAGTATCCTCCTAAAGTCACCTCCTAACGTCCATCCTAAAGCC‑AACACCTAAATCCTACACCTAAAGACCCATCAAGTCAACGCCAATCTTAAAGTTTAAACA‑‑‑‑‑‑‑‑‑‑‑‑‑taaagaccagac                                                                                                                                                                                                           <  1:182229/286‑1 (MQ=255)`  
   `gtgtataagagacaggtACGGACCTAAAGTT‑CCCCCATAGGGGGTACCTAAAGCCCAGCCAATCACCTAAAGTCAACCTTCGGTTGACCTTGAGGGTTCCCTAAGGGTTGGGGATGACCATTGGGTTTGTCTTTGGGTGTTACCTTGAGTGTCTCTCTGTGTCC‑CCTATCTGTTACAGTCTCCTAAAGTATCCTCCTAAAGTCACCTCCTAACGTCCATCCTAAAGCC‑AACACCTAAAGCCTACACCTAAAGACCCATCAAGTCAACGCCTATCTTAAAGTTTAAACA‑‑‑‑‑‑‑‑‑‑‑‑‑taaagaccagacc                                                                                                                                                                                                          <  1:169832/286‑1 (MQ=255)`  
        `agagacaggggtACGGACCTAAAGTT‑CCCCCATAGGGGGTACCTAAAGCCCAGCCAATCACCTAAAGTCAACCTTCGGTTGACCTTGAGGGTTCCCTAAGGGTTGGGGATGACCCTTGGGTTTGTCTTTGGGTGTTACCTTGAGTGTCTCTCTGTGT‑C‑CC‑ATCTGTTACAGTCTCCTAAAGTATCCTCCTAAAGTCACCTCCTAACGTCCATCCTAAAGCC‑AACACCTAAAGCCTACACCTAAAGACCCATCAAGTCAACGCCTATCTTAAAGTTTAAACA‑‑‑‑‑‑‑‑‑‑‑‑‑taaagaccagacc‑taaagac                                                                                                                                                                                                  <  1:295386/291‑1 (MQ=255)`  
          `agcttcaggtgcGGACCTAACGTT‑CCCCCATAGGGGGTACCTACAGCCCAGCCAATCACTTAAAGTCAACCTTCGGTTGACCTTGAGGGTTCCCTAAGGGTTGGGGATGACCCTTGGGTTTGTCTTTGGGTGTTACCTTGAGTGTCTCTCTGTGTCC‑CCTATCTGTTACAGTCTCCTAAAGTATCCTCCTAAAGTCACCTCCTAACGTCCATCCTAAAGCC‑AACACCTAAAGCCTACACCTAAAGACCCATCAAGTCAACGCCTATCTTAAAGTTTAAACA‑‑‑‑‑‑‑‑‑‑‑‑‑taaagaccagacc‑taaagac                                                                                                                                                                                                  <  1:300101/290‑1 (MQ=255)`  
                `gtgtACGGACCTAAAGTT‑CCCCCATAGGGGGTACCTAAAGTCCAGCCAATCACCTAAAGTCAACCTTCGTTTGACCTTGAGGGTTCCCTAAGGGTTGGGGATGACCCTTGGGTTTGTCTTTGGGTGTTAACTTGAGTGTTTTTCTGCGT‑CTCCTATATGTTACAGTCTCCTAAAGTGTCCTCCTAAAGTCACCTCCTAACGTCCATCCTAAAGCC‑AACACCTAAAGCCTACACCTCAAGACTCATCAAGTCAAAGCCTATCTTAAAGTTTAACCA‑‑‑‑‑‑‑‑‑‑‑‑‑taacaaccagacc‑taaagactagaac                                                                                                                                                                                            >  1:342490/1‑299 (MQ=21)`  
                `gtgtACGGACCTAAAGTT‑CCCCCATAGGGGGTACCTAAAGCCCAGCCAATCACCTAAAGTCAACCTTCGGTTGACCTTTAGGGTTCCCTAAGGGTTGGGGATGACCCTTGGGTTTGTCTTTGGGTGTTACCTTGAGTGTCTCTCTTTGTCC‑CCTATCTGTTACAGTCTCCTAAAGTATCCTCCTAAAGTCACCTCCTAACGTCCATCCTAAAGCC‑AACACCTAAAGCCTACACCTAAAGACCCATCAAGTCAACGCCTATCTTAAAGTTTAAACA‑‑‑‑‑‑‑‑‑‑‑‑‑taaagaccagacc‑taaagacctgtct                                                                                                                                                                                            >  1:103786/1‑296 (MQ=255)`  
                `gtgtACGGACCTAAAGTT‑CCCCCATAGGGGGTACCTAAAGCCCAGCCAATCACCTAAAGTCAACCTTCGGTTGACCTTTAGGGTTCCCTAAGGGTTGGGGATGACCCTTGGGTTTGTCTTTGGGTGTTACCTTGAGTGTCTCTCTGTGT‑C‑CCTATCTGTAACAGTCTCCTAAAGTATCCTCCTAAAGTCACCTCCTAACGTCCATCCTAAAGCC‑AACACCTAAAGCCTACACCTAAAGACCCATCAAGTCAAAGCCTATCTTAAAGTTTAAACA‑‑‑‑‑‑‑‑‑‑‑‑‑taaagaccagacc‑taaagaccctgtct                                                                                                                                                                                           >  1:197938/1‑295 (MQ=255)`  
                `gtgtACGGACCTAAAGTT‑CCCCCATAGGGGGTACCTAAAGCCCAGCCAATCACCTAAAGTCAACCTTCGGTTGACCTTGAGGGTTCCCTAATGGTTGGGGATGACCCTTGGGTTTGTCTTTGGGTGTTACCTTGAGTGTCTCTCTGTGT‑C‑CCTATCTGTAACAGTCTCCTAAAGTATCCTCCTAAAGTCACCTCCTAACGTCCATCCTAAAGCC‑AACACCTAAAGCCTACACCTAAAGACCTATCAATTCAACGCCTATCTTAAAGTTTAAACA‑‑‑‑‑‑‑‑‑‑‑‑‑Taaagaccagacc‑taaactccagacct                                                                                                                                                                                           >  1:247082/1‑301 (MQ=255)`  
                `gtgtACGGACCTAAAGTT‑CCCCCATAGGGGGTACCTAAAGCCCAGCCAATCACCTAAAGTCAACCTTCGGTTGACCTTGAGGGTTCCCTAAGGGTTGGGGATGACCCTTGGGTTTGTCTTTGGGTGTTACCTTGAGTGTCTCTCTGTGTCC‑CCTATCTGTTACAGTCTCCTAAAGTATCCTCCTAAAGTCACCTCCTAACGTCCATCCTAAAGCC‑AACACCTAAAGCCTACACCTAAAGACCCATCAAGTCAACGCCTATCTTACAGTTTAAACA‑‑‑‑‑‑‑‑‑‑‑‑‑taaagaccagacc‑taaagaccagaac                                                                                                                                                                                            >  1:42957/1‑299 (MQ=255)`  
                `gtgtACGGACCTAAAGTT‑CCCCCATAGGGGGTACCTAAAGCCCAGCCAATCACCTAAAGTCAACCTTCGGTTGACCTTGAGGGTTCCCTAAGGGTTGGGGATGACCCTTGGGTTTGTCTTTGGGTGTTACCTTGAGTGTCTCTCTGTGT‑C‑CC‑ATCTGTTACAGTCTCCTAAAGTATCCTCCTAAAGTCACCTCCTAACGTCCATCCTAAAGCC‑AATACCTAAAGCCTACACCTAAAGACCCATCAAGTCAACGCCTATCTTAAAGTTTAAACA‑‑‑‑‑‑‑‑‑‑‑‑‑TAaagaccagacc‑taaagaccagaccta                                                                                                                                                                                          >  1:268471/1‑301 (MQ=255)`  
                `gtgtACGGACCTAAAGTT‑CCCCCATAGGGGGTACCTAAAGCCCAGCCAATCACCTAAAGTCAACCTTCGGTTGACCTTGAGGGTTCCCTAAGGGTTGGGGATGACCCTTGGGTTTGTCTTTGGGTGTTACCTTGAGTGTCTCTCTGTCT‑C‑CCTATCTGTAACAGTCTCCTAAAGTATCCTCCTAAAGTCACCTCCTAACGTCCATCCTAAAGCC‑AACACCTAAAGCCTACACCTAAAGACCCATCAAGTCAACGCCTATCTTAAAGTTTAAACA‑‑‑‑‑‑‑‑‑‑‑‑‑Taaagaccagacc‑taaagaccagacct                                                                                                                                                                                           >  1:87625/1‑301 (MQ=255)`  
                `gtgtACGGACCTAAAGTT‑CCCCCATAGGGGGTACCTAAAGCCCAGCCAATCACCTAAAGTCAACCTTCGGTTGACCTTGAGGGTTCCCTAAGGGTTGGGGATGACCCTTGGGTTTGTCTCTGGGTGTTACCTTGAGTGTCTCTCTGTGT‑C‑CCTATCTGTAACAGTCTCCTAAAGTATCCTCCTAAAGTCACCTCCTAACGTCCATCCTAAAGCC‑AACACCTAAAGCCTACACCTAAAGACCCATCAAGTCAACGCCTATCTTAAAGTTTAAACA‑‑‑‑‑‑‑‑‑‑‑‑‑taaagaccacacc‑taaagctgtctctt                                                                                                                                                                                           >  1:55034/1‑292 (MQ=255)`  
                `gtgtACGGACCTAAAGTT‑CCCCCATAGGGGGTACCTAAAGCCCAGCCAATCACCTAAAGTCAACCTTCGGTTGACCTTGAGGGTTCCCTAAGGGTTGGGGATGACACTTGGGTTTGTCTTTGGGTGTTACCTTGAGTGTCTCTCTGTGT‑C‑CCTATCTG‑TACAGTCTCCTAAAGTATCCTCCTAAAGTCACCTCCTAACGTCCATCCTAAAGCC‑AACACCTAAAGCCTACACCTAAAGACCCATCAAGTCAACGCCTATCTTAAAGTTTAAACA‑‑‑‑‑‑‑‑‑‑‑‑‑taaagaccagacc‑taaagctgtctctta                                                                                                                                                                                          >  1:262268/1‑291 (MQ=255)`  
                `gtgtACGGACCTAAAGTT‑CCCCCATAGGGGGTACCTAAAGCCCAGCCAATCACCTAAAGTCAACCTTCGATTGACCTTGAGGGTTCCCTAAGGGTTGGGGATGACCCTTGGGTTTGTCTTTGGGTGTTACCTTGAGTGTCTATATGTGTCC‑CCTATCTGTTTCAGTCTCCTAAAGTATCCTCCTAGAGTCACCTCCTAACGTCCATCCTACAGCC‑AACACCTAAAGACTACACCTAAAGACCCATCAAGGCAACGCCTATCTTAAAGGTTAAACA‑‑‑‑‑‑‑‑‑‑‑‑‑taaagaccagacc‑taaagaccagacc                                                                                                                                                                                            >  1:328820/1‑301 (MQ=25)`  
                `gtgtACGGACCTAAAGTT‑CCCCCATAGGGGGTACCTAAAGCCAAGCCAATCACCTAAAGTCAACCTTCGGTTGACCTTGAGGGTTCCCTAAGGGTTGGGGATGACCCTTGGGTTTGTCTTTGGGTGTTACCTTGAGTGTCTATCTGTGT‑C‑CCTATCTGTAACAGTCTCCTAAAGTATCCTCCTAAAGTCACCTCCTAACGTCCATCCTAAAGCA‑AACACCTAAAGCCTACACCTAAAGACCCATCAAGTCAACGCCTATCTTAAAGTTTAAACA‑‑‑‑‑‑‑‑‑‑‑‑‑Taaagaccagacc‑taaagaccagacct                                                                                                                                                                                           >  1:272831/1‑301 (MQ=255)`  
                `gtgtACGGACCTAAAGTT‑CCCCCATAGCGGGTACCTAAAGCCCAGCCAATCACCTAAAGTCAACCTTCGGTTGACCTTGAGGGTTCCCTAAGGGTTGGGGATGACCCTTGGGTTTTTCTTTGGGTGTTACCTTGAGTGTATCTCTTTGT‑C‑CCTATCTGTAACAGTCTCCTAAAGTATCCTCCTAAAGTCACCTCCTAACGTCCATCCTAAAGCC‑AACACCTAAAGCCTACACCTAAAGACCCATCAAGTCAACGCCTATCTTAAAGTTTAAACA‑‑‑‑‑‑‑‑‑‑‑‑‑Taaagaccagacc‑taacgaccaaacct                                                                                                                                                                                           >  1:155712/1‑301 (MQ=255)`  
                  `gtACGGACCTAAAGTT‑CCCCCATAGGGGGTACCTCAAGCCCAGCCAATCACCTAAAGTCAACCTTCGGTTGACCTTGAGGGTTCCCTAAGGGTTGGGCATGACCCTTGGGTTTGTCTTTGGGTGTTACCTTGCGTGTCTCTCTGTGT‑C‑CCTATCTG‑TACAGTCTCCTAAAGTATCCTCCTAAAGTCACCTTCTAACGTCCATTCTCAAGCC‑ACCTCCTAAAGCCTACACCTAAAGACCCATCCAGCCCACGCCTCTCCTACAGTTTAAACA‑‑‑‑‑‑‑‑‑‑‑‑‑TAAAgaccagaca‑taaagcccagccccaaa                                                                                                                                                                                        >  1:164014/1‑301 (MQ=21)`  
                  `gtACGGACCTAAAGTT‑CCCCCATAGGGGGTACCTAAAGCCCAGCCAATCACCTAAAGTCAACCTTCGGTTGACCTTGAGGGTTCCCTAAGGGTTGGGGATGACCCTTGGGTTTGTCTTTGGGTGTTACCTTGAGTGTCTCTCTGTGTCC‑CCTATCTGTAACAGTCTCCTAAAGTATCCTCCTAAAGTCACCTCCTAACGTCCATCCTAAAGCC‑AACACCTAAAGCCTACACCTAAAGACCAATCAAGTCAACGCCTATCTTAAAGTTTAAACA‑‑‑‑‑‑‑‑‑‑‑‑‑TAaagacaagacc‑taaagaccagaccta                                                                                                                                                                                          >  1:39837/1‑301 (MQ=255)`  
                  `gtACGGACCTAAAGTT‑CCCCCATAGGGGGTACCTAAAGCCCAGCCAATCACCTAAAGTCAACCTTCGGTTGACCTTGAGGGTTCCCTAAGGGTTGGGGATGACCCTTGGGTTTGTCTTTGGGTGTTACCTTGAGTGTCTCTCTGTGT‑C‑CCTATCTGTAACAGTCTCCTAAAGTATCCTCCTAAAGTCACCTCCTAACGTCCATCCTAAAGCC‑AACACCTAAAGCCTACACCTAAAGACCCATCAAGTCAACGCCTATCTTAAAGTTTAAACA‑‑‑‑‑‑‑‑‑‑‑‑‑TAAagaccagacc‑taaagaccagacctaa                                                                                                                                                                                         >  1:278762/1‑301 (MQ=255)`  
                  `gtACGAACCTAAAGTT‑CCCCCATAGGGGGTACCTAAAGCCCAGCCAATCACCTAAAGTCAACCTTCGGTTGACCTTGAGGGTTCCCTAAGGGTTGGGGATGACCCTTGGGTTTGTCTTTGGGTGTTACCTTGAGTGTCTCTCTGTGT‑C‑CC‑ATCTGTTACAGTCTCCTAAAGTATCCTCCTAAAGTCACCTCCTAACGTCCATCCTAAAGCC‑AACACCTAAAGCCTACACCTAAAGACCCATCAAGTAAACGCCTATCTTAAAGTTTAAACA‑‑‑‑‑‑‑‑‑‑‑‑‑TAAAgaccagacc‑taaagacccgacctaaa                                                                                                                                                                                        >  1:159046/1‑301 (MQ=255)`  
                  `atgtgtaaaagagacagcccccATAGGGGGTACCAAATGCCCATCCA‑‑‑AGCTAAAGTCAACCTTCGTTTGACCGTGAGGATTCCCTAAGGGGTGGGGATGACCCGTGGGTTTGTCGTTGGGTGTTACCTTGAGTGTCTCTCTGTGT‑C‑CCTATCTGTAACAGTCTCCTAAAGTAACCTCCTAAAGTCACCTCCTAACGTCCATACTAAAGCC‑AACACCTAAAGCCTACACCTAAAGACCCATCAAGTCAACGCCTATCTTAAAGTTTAAACA‑‑‑‑‑‑‑‑‑‑‑‑‑TAAAGaccagacc‑taaagaccagacctaaag                                                                                                                                                                                       <  1:321421/284‑1 (MQ=37)`  
                    `aCGGACCTAAAGTT‑CCCCCAGAGGGGGTACCTAAAGCCCAGCCAATCACCTAAAGTCAACCTTCGGTTGACCTTGAGGGTTCCCTAAGGGTTGGGGATGACCCTTGGGTTTGTCTTTGGGTGTTACCTTGAGTGTCTCTCTGTGT‑C‑CCTATCTGTAACAGTCTCCTAAAGTATCCTCCTAAAGTCACCTCCTAACGTCCATCCTAAAGCC‑AACACCTAAAGCCTACACCTAAAGACCCATCAAGTCAACGCCTATCTTAAAGTTTAAACA‑‑‑‑‑‑‑‑‑‑‑‑‑TAAAGaccagacc‑taaagaccagacctaaag                                                                                                                                                                                       <  1:133256/301‑1 (MQ=255)`  
                       `gACCTAAAGTTCCCCCCATAGGGTGTACCTAAAGCCCAGCCAATCACCTAAAGTCAACCTTCGGTTGACCTTGAGGGTTCCCTAAGGGTTGGGGATGACCCTTGGGTTTGTCTTTGGGTGTTACCTTGAGTGTCTCTCTGTGT‑C‑CCTATCTG‑TACAGTCTCCTAAAGTATCCTCCTAAAGTCACCTCCTAACGTCCATCCTAAAGCC‑AACACCTAAAGCCTACACATAAAGACCTATCAAGTCAACGCCTATCTTAAAGTTTAAACA‑‑‑‑‑‑‑‑‑‑‑‑‑TAAAGACCAGACA‑TAAAGACCAGACCTAAAGaca                                                                                                                                                                                    >  1:117644/1‑301 (MQ=255)`  
                       `gACCTAAAGTT‑CCCCCATAGGGGGTACCTAAAGCCCAGCCAATCACCTAAAGTCAACCTTCTGTTGACCTTGTGGGTTCCCTAAGGGTTGTGGATGACCCTTGGGTTTGTCTTTGGGTGTTACCTTGAGTGTCTCTCTGTGT‑C‑CCTATCTGTAACAGTCTCCTAAAGTATCCTCCTAAAGTCACCTCCTAACGTCCATCCTAAAGCC‑AACACCTCAAGCCTACACCTAAAGACCCATCAAGTCAACGCCTATCTTAACGTTTAACCA‑‑‑‑‑‑‑‑‑‑‑‑‑TAAAGACCCTACC‑TAAAGCCCAGCCCTAAAGaca                                                                                                                                                                                    >  1:313061/1‑301 (MQ=25)`  
                       `gACCTAAAGTT‑CCCCCATAGGGGGTACCTAAAGCCCAGCCAATCACCTAAAGTCAACCTTCGGTTGACCTTGAGGGTTCCCTAAGGGTTGGGGATGACCCTTGGGTTTGTCTTTGGGTGTTACCTTGAGTGTCTCTCTGTGT‑C‑CCTATCTG‑TACAGTCTCCTAAAGTATCCTCCTAAAGTCACCTCCTAACGTCCATCCTAAAGCC‑AACACCTAAAGCCTACACCTAAAGACCCATCAAGTCAACGCCTATCTTAAAGTTTAAACA‑‑‑‑‑‑‑‑‑‑‑‑‑TAAAGACCAGACC‑TAAAGACCAGACCTAAAGacac                                                                                                                                                                                   >  1:304012/1‑301 (MQ=255)`  
                       `gACCTAAAGTT‑CCCCCATAGGGGGTACCTAAAGCCCAGCCAATCACATAACGTCAACCTTCGGTTGACCTTGAGGGTTCCCTAAGGGTTTGGGATGACCCTTGGGTTTGTCGTTGGGTGTTACCTTGAGTGTCTCTCTGTGT‑C‑CC‑ATCTTTTACAGTCTCCTAAAGTATCCTCCTAAAGTAACCTCCTAACGTCCATCCTAAAGCC‑AACACCTAAAGCCTACACCTAAAGACCCATCAAGTCAACGCCTATCTTAAAGTTTAAACA‑‑‑‑‑‑‑‑‑‑‑‑‑TAACGACCAGACC‑TAAAGACCATACCTAAAGacac                                                                                                                                                                                   >  1:152135/1‑301 (MQ=255)`  
                         `ccTAAAGTT‑CCCCCATAGGGGGTACCTAAAGCCCAGCCAATCACCTAAAGTCAACCTTCGGTTGACCTTGAGGGTTCCCTAAGGGTTGGGGATGACCCTTGTGTTTGTCTTTGGGTGTTACCTTGAGTGTATCTCTGTGTCC‑CCTATCTGTTACAGTCTCCTAAAGTATCCTCCTAAAGTCACCTCCTAACGTCCATCCTAAAGCC‑AACACCTAAAGCCTACACCTAAAGACCCATCAAGTCAACGCCTATCTTAAAGTTTAACCA‑‑‑‑‑‑‑‑‑‑‑‑‑TAAAGACCAAACC‑TACAGACCAGACCTAAAGacac                                                                                                                                                                                   >  1:48487/1‑301 (MQ=255)`  
                                `tt‑CCCCCATAGGGGGTACCTAAAGCCCAGCCAATCACCTAAAGTCAACCTTCGGTTGACCTTGAGGGTTCCCTAAGGGTTGGGGATGACCCTTGGGTTTGTCTTTGGGTGTTACCTTGAGTGTCTCTCTGTGT‑C‑CCTATCTGTAACAGTCTCCTAAAGTATCCTCCTAAAGTCACCTCCTAACGTCCATCCTAAAGCC‑AACACCTAAAGCCTACACCTAAAGACCCATCAAGTCAACGCCTATCTTAAAGTTTAAACA‑‑‑‑‑‑‑‑‑‑‑‑‑TAAAGACCAGAAC‑TAAAGACCAGACCTAAAGACACTACATaaa                                                                                                                                                                           <  1:259371/301‑1 (MQ=255)`  
                                   `gtgtataagagacagccTAAAGCCCAGCCAATCACCTAAAGTCAACCTTCGGTTGACCTTGAGGGTTCCCTAAGGGTTGGGGATGACCCTTGGGTTTGTCTTTGGGTGTTACCTTGAGTGTCTCTCTGTGT‑C‑CC‑ATCTGTTACAGTCTCCTAAAGTATCCTCCTAAAGTCACCTCCTAACGTCCATCCTAAAGCC‑AACACCTAAAGCCTACACCTAAAGACCCATCAAGTCAACGCCTATCTTAAAGTTTAAACA‑‑‑‑‑‑‑‑‑‑‑‑‑TAAAGACCAGACC‑TAAAGACCAGACCTAAAGACACTACATAAagac                                                                                                                                                                        <  1:296982/286‑1 (MQ=255)`  
                                   `cccccATAGGGGGTACCTAAAGCCCAGCCAATCACCT‑AAGTCAACCTTCGGTTGACCTTGAGGGTTCCCTAAGGGTTGGGGATGACCCTTGGGTTTGTCTTTGGGTGTTACCTTGAGTGTCTCTCTGTGT‑C‑CCTATCTGTAACAGTCTCCTAAAGTATCCTCCTAAAGTCACTTCCTAACGTCCATCCTAAAGCC‑AACACCTAAAGCCTACACCTAAAGACCCATCAAGTCAACGCCTATCTTAAAGTTTAAACA‑‑‑‑‑‑‑‑‑‑‑‑‑TAAAGACCAGACC‑TAAAGACCAGACCTAAAGACACTACATAAagac                                                                                                                                                                        <  1:83648/301‑1 (MQ=255)`  
                                   `cccccATAGGGGGTACCTAAAGCCCAACCAAACACCTAAATTCAACCTTCGTGTGACCTTGAGGGTTCCCTAAGGGTTTGGGATGACCCTTGGGTTTGTCGTTGGGTGTTACCTTGAGTGTCTCTCTTTGT‑C‑CCTATCTG‑TACAGTCTCCTAAAGTATCCTCCTAAAGTCACCTCATAACGTCCATCCTAAAGCC‑AACACCTAAAGCCTACACCTAAAGACCCATCAAGTCAACGCCTATCTTAAAGTTTAAACA‑‑‑‑‑‑‑‑‑‑‑‑‑TAAAGACCAGACC‑TAAAGACCAGACCTAAAGACAATACATAAagac                                                                                                                                                                        <  1:332974/301‑1 (MQ=255)`  
                                    `gagacaggggggTACCTAAAGCCCAGCCAATCACCTAAAGTCAACCGTCGGTTGACCTTGAGGGTTCCCTAAGGGTTGGGGATGACCCTTGGGTTTGTCTTTGGGTGTTACCTTGAGTGTCTCTCTGTGC‑C‑CCTATCTGTTACAGTCTCCTAAAGTATCCTCCTAAAGTCACCTCCTAACGTCCATCCTAAAGCC‑AACACCTAAAGCCTACACCTAAAGACCCATCAAGTCAACGCCTATCTTAAAGTTTAAACA‑‑‑‑‑‑‑‑‑‑‑‑‑TAAAGACCAGACC‑TAAAGACCAGACCTAAAGACACTACATAAagac                                                                                                                                                                        <  1:96954/294‑1 (MQ=255)`  
                                     `acagtAGGGTGTACCTAAAGCCCAGCCAATCACCTAATGTCAACCTTCGGTTGACCTTGAGGGTTCCCTAATGGTTGGGGATGACCCGTGGGTTTGTCTTTGGGTGTTACCTTGAGTGTCTCTCTGTGT‑C‑CCTATCTGTAACAGTCTCCTAAAGTATCCTCCTAAAGTCACCTCCTAACGTCCATCCTAAAGCC‑AACACCTAAGGCCTACACCTAAAGACCCATCAAGTCAACGCCTATCTTAAAGTTTAAACA‑‑‑‑‑‑‑‑‑‑‑‑‑TAAAGACCAGACC‑TAAAGACCAGACCTAAAGACACTACATAAagacc                                                                                                                                                                       <  1:289682/297‑1 (MQ=255)`  
                                      `ccATAGTGGGTACCTAAAGCCCAGCCAATCACCTAAAGTCAACCTTCGGTTGACATTGAGGGTTCCCTAAGGGTTGGGGATGACCCTTGGGTTTGTCTTTGGGTGTTACCTTGAGTGTCTCTCTGTGT‑C‑CCTATCTG‑TACAGTCTCCTAAAGTATCCTCCTAAAGTCACCTCCTAACGTCCATCCTAAAGCC‑AACACCTAAAGCCTACACCTAAAGACCCATCAAGTCAACGCCTATCTTAAAGTTTAAACA‑‑‑‑‑‑‑‑‑‑‑‑‑TAAAGACCAGACC‑TAAAGACCAGACCTAAAGACACTACATAAagaccaa                                                                                                                                                                     >  1:205984/1‑300 (MQ=255)`  
                                      `ccATAGGGGGTACCTAAAGCCCAGCCAATCACTTAAAGTCAACCTTCGGTTGACCTTGAGGGTTCCCTAAGGGTTGGGGATGACCCTTGGGTTTGTCTTTGGGTGTTACCTTGAGTGTCTCTCTGTGTCC‑CCTATCTGTTACAGTCTCCTAAAGTATCCTCCTAAAGTCACCTCCTAACGTCCATCCTAAAGCC‑AACACCTAAAGCCTACACCTAAAGACCCATCAAGTCAACGCCTATCTTCACGTTTAAACA‑‑‑‑‑‑‑‑‑‑‑‑‑TAAAGACCAAACC‑TAAAGACCAGACCTAAAGACACTACATAAagacc                                                                                                                                                                       >  1:61535/1‑301 (MQ=255)`  
                                        `aTAGGGGGTACCTAAAGCCCAGCCAATCACCTAAAGTCAACCTTCGGTTGACCTTGAGGGTTCCCTAAGGGTTGGGGATGACCCTTGGGTTTGTCTTTGGGTGTTACCTTGAGTGTCTCTCTGTGT‑C‑CCTATCTGTAACAGTCTCCTAAAGTATCCTCCTAAAGTCACCTCCTAACGTCCATCCTAAAGCC‑AACACCTAAAGCCTACACCTAAAGACCCATCAAGTCAACGCCTATCTTAAAGTTTAAACA‑‑‑‑‑‑‑‑‑‑‑‑‑TAAAGACcagacc‑taaagaccagacctaaagacctgtctcttatacacat                                                                                                                                                                    >  1:92395/1‑284 (MQ=255)`  
                                             `gggTACCTAAAGCCCAGCCAATCACCTAAAGTCAACCTTCGGTTGACCTTGAGGGTTCCCTAAGGGTTTGGGATGACCCTTTGGTTTGTCTTTTGGTGTTACCTTGAGAGTCTCTCTGTGT‑C‑CC‑ATCTGTTACAGTCTCCTAAAGTATCCTCCTAAAGTCACCTCCTAACGTCCATCCTAAAGCC‑AACACCTAAAGCCTACACCTAAAGACCCATCAAGTCAACGCCTATCTTAAAGTTTAAACA‑‑‑‑‑‑‑‑‑‑‑‑‑TAAAGACCAGACC‑TAAAGACCAGACCTAAAGACACTACATAAAGACCAGACCTaaa                                                                                                                                                              >  1:144669/1‑301 (MQ=255)`  
                                             `gggTACCTAAAGCCCAGCCAATCACCTAAAGTCAACCTTCGGTTGACCTTGAGGGTTCCCTAAGGGTTGGGGATGACCCTTGGGTTTGTCTTTGGGTGTTACCTTGAGTGTCTCTCTGTGT‑C‑CCTATCTGTAACAGTCTCCTAAAGTATACTCCTAAAGTCACCTCCTAACGTCCATACTAAAGCC‑AACACCTAAAGCCTACACCTAAAGACCCATCAAGTCAACGCCTATCTTAAAGTTTAAACA‑‑‑‑‑‑‑‑‑‑‑‑‑TAAAGACCAGACC‑TAAAGACCAGACCTAAAGacacctgtctcttatacacatctc                                                                                                                                                               >  1:276690/1‑281 (MQ=255)`  
                                             `gggTACCTAAAGCCCAGCCAATCACCTAAAGTCAACCTTCGGTTGACCTTGAGGGTTCCCTAAGGGTTGGGGATGACCCTTGGGTTTGTCTTTGGGTGTTACCTTGAGTGTCTCTCTGTGT‑C‑CC‑ATCTGTTACAGTCTCCTAAAGTATCCTCCTAAAGTCACCTCCTAACGTCCATCCTAAAGCC‑AACACCTAAAGCCTACACCTAAAGACCCATCAAGTCAACGCCTATCTTAAAGTTTAAACA‑‑‑‑‑‑‑‑‑‑‑‑‑TAAAGACCAGACC‑TAAAGACCAGACCTAAAGACACTACATAAAGACCAGACCTaaa                                                                                                                                                              >  1:130443/1‑301 (MQ=255)`  
                                                 `aCCTAAAGCCCAGCCAATCACCTAAAGTCAACCTTCGGTTGACCTTGAGGGTTCCCTAAGGGTTGGGGATGACCCTTGGGTTTGTCTTTGGTTGTTACCTTGAGTGTCTCTCTGTGT‑C‑CCTATCTGTAACAGTCTCCTAAAGTATCCTCCTAAAGTCACCTCCTAACGTCCATCCTAAAGCC‑AACACCTAAAGCCTACACCTAAAGACCCATCAAGTCAACGCCTATCTTAAAGTTTAAACA‑‑‑‑‑‑‑‑‑‑‑‑‑TAAAGACCAGACC‑TAAAGACCAGACCTAAAGACACTACATAAagctgtctcttatacac                                                                                                                                                           >  1:256552/1‑286 (MQ=255)`  
                                                 `aCCTAAAGCCCAGCCAATCACCTAAAGTCAACCTTCGGTTGACCTTGAGGGTTCCCTAAGGGTTGGGGATGACCCTTGGGTTTGTCTTTGGGTGTTACCTTGAGTGTCTCTCTGTGTCC‑CCTATCTGTTACAGTCTCCTAAAGTATCCTCCTAAAGTCACCTCCTAACGTCCATCCTAAAGCC‑AACACCTAAAGCCTACACCTATAGACCCATCAAGTCAACGCCTATCTTAAAGTTTAAACA‑‑‑‑‑‑‑‑‑‑‑‑‑TAAAGACCAGACC‑TAAAGACCAGACCTAAAGACACTACATAAagactgtctcttatac                                                                                                                                                            >  1:18551/1‑289 (MQ=255)`  
                                                       `aGCCCAGCCAATCACCTAAAGTCAACCTTCGGTTGACCTTGAGGGTTCCCTAAGGGTTGGGGATGACCCTTGGGTTTGTCTTTGGGTGTTACCTTGAGTGTCTCTCTGTGT‑C‑CCTATCTGTAACAGTCTCCTAAAGTATCCTCCTAAAGTCACCTCCTAACGTCCATCCTAAAGCC‑AACACCTAAAGCCTACACCTAAAGACCCATCAAGTCAACGCCTATCTTAAAGTTTAAACA‑‑‑‑‑‑‑‑‑‑‑‑‑TAAAGACCAGACC‑TAAAGACCAGACCTAAAGACACTACATAAAGACCAGACCTAAAGACGCCttt                                                                                                                                                     >  1:221091/1‑300 (MQ=255)`  
                                                        `gCCCAGCCAATCACCTAAAGTCAACCTTCGGTTGACCTTGAGGGTTCCCTAAGGGTTGGGGATGACCCTTGGGTTTGTCTTTGGGTGTTACCTTGAGTGTCTCTCTGTGT‑C‑CCTATCTG‑TACAGTCTCCTAAAGTATCCTCCTAAAGTCACCTCCTAACGTCCATCCTAAAGCC‑AACACCTAAAGCCTACACCTAAAGACCCATCAAGTCAACGCCTATCTTAAAGTTTAAACA‑‑‑‑‑‑‑‑‑‑‑‑‑TAAAGACCAGACC‑TAAAGACCAGACCTAAAGACACTACATAAagaccagaccctgtctgttatacac                                                                                                                                                   >  1:126943/1‑286 (MQ=255)`  
                                                              `ccAATCACCTAAAGTCAACCTTCGGTTGACCTTGAGGGTTCCCTAAGGGTTGGGGATTACCCTTGGGTTTGTCTTTGGGTGTTACCTTGAGTGTCTCTCTGTGT‑C‑CCTATCTG‑TACAGTCTCCTAAAGTATCCTCCTAAAGTCACCTCCTAACGTCCATCCTAAAGCC‑AACACCTAAAGCCTACACCTAAAGACCCATCAAGTCAACGCCTATCTTAAAGTTTAAACA‑‑‑‑‑‑‑‑‑‑‑‑‑TAAAGACCAGACC‑TAAAGACCAGACCTAAAGACACTACATAAAGACCAGACCTAAAGACGCCTTGTTGTTAGc                                                                                                                                             <  1:23812/301‑1 (MQ=255)`  
                                                                   `cACCTAAAGTCAACCTTCGGTTGACCTTGAGGGTTCCCTAAGGGTTGGGGATGACCCTTGGGTTTGTCTTTGGGTGTTACCTTGAGTGTCTCTCTGTGT‑C‑CC‑ATCTGTTACAGTCTCCTAAAGTATCCTCCTAAAGTCACCTCCTAACGTCCATCCTAAAGCC‑AACACCTAAAGCCTACACCTAAAGACCCATCAAGTCAACGCCTATCTTAAAGTTTAAACA‑‑‑‑‑‑‑‑‑‑‑‑‑TAAAGACCAGACC‑TAAAGACCAGACCTAAAGACACTACATAAAGACCAGACCTaaaactgactctaatacacatccac                                                                                                                                        >  1:208415/1‑279 (MQ=255)`  
                                                                       `aaaaGTCAACCTTCGGGTGACATTGAGGGTTCCCTAAGGGTTGGGGATGACCCTTGGGTTTGTCTTGGGGTGTTCCCTTGAGTGTCTCTCTGTGTCC‑CCTATCTGTTACAGTCTCCTAAAGTATCCTCCTAAAGTCACCTCCTAACGTCCATCCTAAAGCC‑AACACCTAAAGCCTACACCTAAAGACCCATCAAGTCAACGCCTATCTTAAAGTTTAAACA‑‑‑‑‑‑‑‑‑‑‑‑‑TAAAGACCAGACC‑TAAAGACCAGACCTAAAGACACTACATAAAGACCAGACCTAAAGACGCCTTGTTGTTAGCCATAAAg                                                                                                                                      <  1:204008/300‑1 (MQ=255)`  
                                                                            `tCAACCTTCGGTTGTCCTTGAGGGTTCCCTAAGGGTTGGGGATGACCCTTGGGTTTGTCTTTGGGTGTTACCTTGAGTGTCTCTCTGTGT‑C‑CCTATCTGTAACAGTCTCCTAAAGTATCCTCCTAAAGTCACCTCCTAACGTCCATCCTAAAGCC‑AACACCTAAAGCCTACACCTAAAGACCCATCAAGTCAACGCCTATCTTAAAGTTTAAACA‑‑‑‑‑‑‑‑‑‑‑‑‑TAAAGACCAGACC‑TAAAGACCAGACCTAAAGACCAGACCTAAAGACACTACATAAAGAccagacctaaagacgccttgttgttagc                                                                                                                                <  1:2868/301‑28 (MQ=37)`  
                                                                             `cAACCTTCGGTTGACCTTGAGGGTTCCCTAAGGGTTGGGGATGACCCTTGGGTTTGTCTTTGGGTGTTACCTTGAGTGTCTCTCTGTGT‑C‑CC‑ATCTGTTACAGTCTCCTAAAGTATCCTCCTAAAGTCACCTCCTAACGTCCATCCTAAAGCC‑AACACCTAAAGCCTACACCTAAAGACCCATCAAGTCAACGCCTATCTTAAAGTTTAAACA‑‑‑‑‑‑‑‑‑‑‑‑‑TAAAGACCAGACC‑TAAAGACCAGACCTAAAGACACTACATAAAGACCAGACCTAAAGACGCCTTGTTGTTAGCCATAAAGTGATAAcc                                                                                                                              <  1:173902/301‑1 (MQ=255)`  
                                                                               `aCCTTCGGTTGACCTTGAGGGTTCCCTAAGGGTTGGGGATGACCCTTGGGTTTGTCTTTGGGTGTTACCTTGAGTGTCTCTCTGTGT‑C‑CCTATCTGTAACAGTCTCCTAAAGTATCCTCCTAAAGTCACCTCCTAACGTCCATCCTAAAGCC‑AACACCTACAGCCTACACCTAAAGACCCATCAAGTCAACGCCTATCTTAAAGTTTAAACA‑‑‑‑‑‑‑‑‑‑‑‑‑TAAAGACCAGACC‑TAAAAACCAGACCTAAAGACACTACATAAAGACCAGACCTAAAGACGCCTTGTTGTTAGCCATAAAGTGATAAccc                                                                                                                             >  1:338266/1‑300 (MQ=255)`  
                                                                                `ccTTCGGTTGACCTTGAGGGTTCCCTAAGGGTTGGGGATGACCCTTGGGTTTGTCTTTGGGTGTTACCTTGAGTGTCTCTCTGTGTCC‑CCTATCCGTTACAGTCTCCTAAAGTATCCTCCTAAAGTCACCTCCTAACGTCCATCCTAAAGCC‑AACACCTAAAGCCTACACCTAAAGACCCATCAAGTCAACGCCTATCTTAAAGTTTAAACA‑‑‑‑‑‑‑‑‑‑‑‑‑TAAAGACCAGACC‑TAAAGACCAGACCTAAAGACACTACATAAAGACCAGACCTAAAGACGCCTTGTTGTTAGCCATAAAGTGATAACCt                                                                                                                             <  1:115382/301‑1 (MQ=255)`  
                                                                                    `ggggtGACCGTGAGGGTTCCCTAAGGGTTGGGGATGACCCTTGGGTTTGTCTTTGGGTGTTACCTTGAGTGTCTCTCTGTGT‑C‑CC‑ATCTGTTACAGTCTCCTAAAGTATCCTCCTAAAGTCACCTCCTAACGTCCATCCTAAAGCC‑AACACCTAAAGCCTACACCTAAAGACCCATCAAGTCAACGCCTATCTTAAAGTTTAAACA‑‑‑‑‑‑‑‑‑‑‑‑‑TAAAGACCAGACC‑TAAAGACCAGACCTAAAGACACTACATAAAGACCAGACCTAAAGACGCCTTGTTGTTAGCCATAAAGTGATAACCTTTAATc                                                                                                                       <  1:73577/297‑1 (MQ=255)`  
                                                                                    `cGGTTGACCTTGAGGGTTCCCTAAGGGTTGGGGATGACCCTTGGGTTTGTCTTTGGGTGTTACCTTGAGTGTCTCTCTGTGT‑C‑CC‑ATCTGTTACAGTCTCCTAAAGTATCCTCCTAAAGTCACCTCCTAACGTCCATCCTAAAGCC‑AACACCTAAAGCCTACACCTAAAGACCCATCAAGTCAACGCCTATCTTAAAGTTTAAACA‑‑‑‑‑‑‑‑‑‑‑‑‑TAAAGACCAGACC‑TAAAGACCAGACCTAAAGACACTACATAAAGACCAGACCTAAAGACGCCTTGTTGTTAGCCATAAAGTGATAACCTTTAATc                                                                                                                       <  1:192229/301‑1 (MQ=255)`  
                                                                                    `cGGTTGACCGTGAGGTTTCCCAGAGGGGTGGGGATTACCCTTGGGTTTGTATTTGGGTGTTACCTTACGTGTTTCTCTGTGT‑A‑AC‑ATCTGATACAGTCGCCTAAAGCAGCCTCCTCGAGGCACCTCCTAACGTCTATGCTAAAGCC‑AACACCTACAGCCTAGACATAAAGACCCACCAGGGCAACGCCTATCTTAAAGTTTAAACA‑‑‑‑‑‑‑‑‑‑‑‑‑TAAAGACCAGACC‑TAAAGACCAGGCCGAAAGACACTAAATAAAGACCAGACCTAAAGACGCCTTGTTGTTAGCCATAAAGTGATAACCTTTAATc                                                                                                                       <  1:201426/301‑1 (MQ=255)`  
                                                                                     `tgTTGACCGTGAGGGTTCCCTAAGGGTTGGGGATGACCCTTGGGTTTGTCTTTGGGTGTTACCTTGAGTGTCTCTCTGTGT‑C‑ACTATCTTTAACAGTCTCCTAAAGTATCCTCCTAAAGTCACCTCCTAACGTCCATCCTAAAGAC‑AACACCTAAAGCCTACACCTAAAGACCCATCAAGTCAACGCCTATCTTAAAGTTTAAACA‑‑‑‑‑‑‑‑‑‑‑‑‑TAAAGACCAGACC‑TAAAGACCAGACCTAAAGACACTACATAAAGACCAGACCTAAAGACGCCTTGTTGTTAGCCATAAAGTGATAACCTTTAATc                                                                                                                       <  1:100116/300‑1 (MQ=255)`  
                                                                                     `gggtGACCTTGAGTGTTCCCTAAGGGTTTGGGATGAACCTTGGGTTTGTCTTTGGGTGTTACCTTGAGTGGCTCTCTGTGT‑C‑CCTATCTGTAACAGTCTACTAAAGTATCCTACTAAAGTCACCTCCTAACGTCCATCCTAAAGCC‑AACACCTAAAGCCTACACCTAAAGACCCATCAAGTCAACGCCTATCTTAAAGTTTAAACA‑‑‑‑‑‑‑‑‑‑‑‑‑TAAAGACCAGACC‑TAAAGACCAGACCTAAAGACACTACATAAAGACCAGACCTAAAGACGCCTTGTTGTTAGCCATAAAGTGATAACCTTTAATc                                                                                                                       <  1:310341/298‑1 (MQ=255)`  
                                                                                      `ttttcttcagcacccccccTATGGGTTGGGGTTGCCCTCTGTGTTTGTCTTTGTGTTTTCCCTGATTTGTCTCCTTTTTC‑C‑CCTTTCCTTTTCCGTCCTCCAAAACATTCTCCTTAATGCCCTCCCTAACCTTCAACCTTACGCCAAACCCTTAAACCTTACTCCAAAATCCCCACAAATTCAAC‑CCCAACTTAAAAGTTTAACC‑‑‑‑‑‑‑‑‑‑‑‑‑TAAAAGCCCGACCTTAATGCCCAG‑CCGTAAGACACTACATAAAGACCAGACCTAAAGACGCCTTGTTGTTAGCCATAAAGTGATAACCTTTAATCa                                                                                                                      <  1:244554/285‑1 (MQ=255)`  
                                                                                      `tgtgtataagagacagcccTAAGGGTTGGGTATGACCCTTGGGTTTGTCTTTGGGTGTTACCTTGAGTGTCTCTCTGTGTCC‑CCTATCTGTTACAGTCTCCTAAAGTATCCTCCTAAAGTCACCTCCTAACGTCCATCCTAAAGCC‑AACACCTAAAGCCTACACCTAAAGACCCATCAAGTCAACGCCTATCTTAAAGTTTAAACA‑‑‑‑‑‑‑‑‑‑‑‑‑TAAAGACCAGACC‑TAAAGACCAGACCTAAAGACACTACATAAAGACCAGACCTAAAGACGCCTTGTTGTTAGCCATAAAGTGATAACCTTTAATc                                                                                                                       <  1:27101/285‑1 (MQ=255)`  
                                                                                                `ggacagcccTAAGTGTTGGGGATGACCCTTGGGTTTGTCTTTGGGTGTTACCTTGAGTGTCGCTCTGTTTCC‑CCTATCTGTTACAGTCTCCTAAAGTATCCTCCTAAAGTCACCACCTAACGTCCATCCTAAAGCC‑AACACCTAAAGCCTACACCTAAATACCCATCAAGTCAACGCCTATCTTAAAGTTTAAACC‑‑‑‑‑‑‑‑‑‑‑‑‑TAAAGACCAGACC‑TAAAGACCAGACCTAAAGACACTACATAAAGACCAGACCTAAAGACGCCTTGTTGTTAGCCATAAAGTGATAACCTTTAATCATTGTCTtta                                                                                                             <  1:162682/295‑1 (MQ=255)`  
                                                                                                 `gggTTCCCTAAGGGTTGGGGATGACCCTTGGGTTTGTCTTTGGGTGTTACCTTGAGTGTCTCTCTGTGT‑C‑CCTATCTGTAACAGTCTCCTAAAGTATCCTCCTAAAGTCACCTCCTAACGTCCATCCTAAAGCC‑AACACCTAAAGCCTACACCTAAAGACCCATCAAGTCAACGCCTATCTTAAAGTTTAAACA‑‑‑‑‑‑‑‑‑‑‑‑‑TAAAGACCAGACC‑TAAAGACCAGACCTAAAGACACTACATAAAGACCAGACCTAAAGACGCCTTGTTGTTAGCCATAAAGTGATAACCTTTAATCATTGTCTttatt                                                                                                           >  1:57702/1‑301 (MQ=255)`  
                                                                                                 `acagtCCCTAAGGGTTGGGGATGACCCTTGGGTTTGTATTTGGGTGTTACCTTGAGTGTCTCTCTGTGT‑C‑CCTATCTGTAACAGTCTCCTAAAGTATCCTCCTAAAGTCACCTCCTAACGTCCATCCTAAAGCC‑AACACCTAAAGCCTACACCTAAAGACCCATCAAGTCAACGCCTATCTTAAAGTTTAAACA‑‑‑‑‑‑‑‑‑‑‑‑‑TAAAGACCAGACC‑TAAAGACCAGACCTAAAGACACTACATAAAGACCAGACCTAAAGACGCCTTGTTGTTAGCCATAAAGTGATAACCTTTAATCATTGTCTttatt                                                                                                           <  1:166203/297‑1 (MQ=255)`  
                                                                                                      `ctcTAAGGGTTGGGGATGACCCTTGGGTTTGTCTTTGGGTGTTACCTTGAGTGTCTCTCTGTGT‑C‑CCTATCTGTAACAGTCTCCTAAAGTATCCTCCTAAAGTCACCTCCTAACGTCCATCCTAAAGCC‑AACACCTAAAGCCTACACCTAAAGACCCATCAAGTCAACGCCTATCTTAAAGTTTAAACA‑‑‑‑‑‑‑‑‑‑‑‑‑TAAAGACCAGACC‑TAAAGACCAGACCTAAAGACACTACATAAAGACCAGACATAAAGACGCCTTGTTGTTAGCCATAAAGTGATAACCTTTAATcctgtctcttataaacat                                                                                                      >  1:176143/3‑284 (MQ=255)`  
                                                                                                           `aGGGTTGGGGATGACCCTTGGGTTTGTCTTTGGGTGTTACCTTGAGTGTCTCTCTGTGT‑C‑CCTATCTGTAACAGTCTCCTAAAGTATCCTCCTAAAGTCACCTCCTAACGTCCATCCTAAAGCC‑AACACCTAAAGCCTACACCTAAAGACCCATCAAGTCAACGCCTATCTTAAAGTTTAAACA‑‑‑‑‑‑‑‑‑‑‑‑‑TAAAGACCAGACC‑TAAAGACCAGACCTAAAGACACTACATAAAGACCAGACCTAAAGACGCCTTGTTGTTAGCCATAAAGTGATAACCTTTAATCATTGTCTTTATTAATACAACTc                                                                                                 <  1:120654/301‑1 (MQ=255)`  
                                                                                                            `gggTTTGGGATGACCCTTGGGTTTGTCTTTTGGTGTTACCTTGAGTGTCTCTCTGTGT‑C‑CCTATCTGTAACAGTCTCCTAAAGTATCCTCCTAAAGTCACCTCCTAACGTCCATCCTAAAGCC‑AACACCTAAAGCCTACACCTAAAGACCCATCAAGTCAACGCCTATCTTAAAGTTTAAACA‑‑‑‑‑‑‑‑‑‑‑‑‑TAAAGACCAGACC‑TAAAGACCAGACCTAAAGACACTACATAAAGACCAGACCTAAAGACGCCTTGTTGTTAGCCATAAAGTGATAACCTTTAATCATTGTCTTTATTAATACAACTcc                                                                                                >  1:112365/1‑300 (MQ=255)`  
                                                                                                            `gggTTGGGGATGACCCTTGGGTTTGTCTTTGGGTGTTACCTTGAGTGTCTCTCTGTGT‑C‑CCTATCTGTAACAGTCTCCTAAAGTATCCTCCTAAAGTCACCTCCTAACGTCCATCCTAAAGCC‑AACACCTAAAGCCTACACCTAAAGACCCATCAAGTCAACGCCTATCTTAAAGTTTAAACA‑‑‑‑‑‑‑‑‑‑‑‑‑TAAAGACCAGACC‑TAAAGACCAGACCTAAAGACACTACATAAAGACCAGACCTAAAGACGCCTTGTTGTTAGCCATAAAGTGATAACCTTTAATCATTGTCTTTATTAATACAACTCa                                                                                                >  1:64277/1‑301 (MQ=255)`  
                                                                                                             `ggTTGGGGATGACCCTTGGGTTTGTCTTTGGGTGTTACCTTGAGTGTCTCTCTGTGT‑C‑CCTATCTGTAACAGTCTCCTAAAGTATCCTCCTAAAGTCACCTCCTAACGTCCATCCTAAAGCC‑AACACCTAAAGCCTACACCTAAAGACCCATCAAGTCAACGCCTATCTTAAAGTTTAAACA‑‑‑‑‑‑‑‑‑‑‑‑‑TAAAGACCAGACC‑TAAAGACCAGACCTAAAGACACTACATAAAGACCAGACCTAAAGACGCCTTGTTGTTAGCCATAAAGTGATAACCTTTAATCATTGTCTTTATTAATACAACTCAc                                                                                               >  1:226983/1‑301 (MQ=255)`  
                                                                                                                `gagatgtgtataagagacaggTCTTTGGGTGTTACCTTGAGTGTCTCTCTGTGTCC‑CCTATCTGTTACAGTCTCCTAAAGTATCCTCCTAAAGTCACCTCCTAACGTCCATCCTAAAGCC‑AACACCTAAAGCCTACACCTAAAGACCCATCAAGTCAACGCCTATCTTAAAGTTTAAACA‑‑‑‑‑‑‑‑‑‑‑‑‑TAAAGACCAGACC‑TAAAGACCAGACCTAAAGACACTACATAAAGACCAGACCTAAAGACGCCTTGTTGTTAGCCATAAAGTGATAACCTTTAATCATTGTCTTTATTAATACAACTCACta                                                                                             <  1:252982/281‑1 (MQ=255)`  
                                                                                                                 `ggggATGACCCTTGGGTTTGTCTTTGGGTGTTACCTTGAGTGTCTCTCTGTGT‑C‑CCTATCTGTAACAGTCTCCTAAAGTATCCTCCTAAAGTCACCTCCTAACGTCCATCCTAAAGCC‑AACACCTAAAGCCTACACCTAAAGACCCATCAAGTCAACGCCTATCTTAAAGTTTAAACA‑‑‑‑‑‑‑‑‑‑‑‑‑TAAAGACCAGACC‑TAAAGACCAGACCTAAAGACACTACATAAAGACCAGACCTAAAGACGCCTTGTTGTTAGCCATAAAGTGATAACCTTTAATCATTGTCTTTATTAATAcctgtctcttat                                                                                           >  1:31637/1‑290 (MQ=255)`  
                                                                                                                   `agacagcccTTGGGTTTGTCTTTGGGTGTTACCTTGAGTGTCTCTCTGTGT‑C‑CCTATCTGTAACAGTCTCCTAAAGTATCCTCCTAAAGTCACCTCCTAACGTCCATCCTAAAGCC‑AACACCTAAAGCCTACACCTAAAGACCCATCAAGTCAACGCCTATCTTAAAGTTTAAACA‑‑‑‑‑‑‑‑‑‑‑‑‑TAAAGACCAGACC‑TAAAGACCAGACCTAAAGACACTACATAAAGACCAGACCTAAAGACGCCTTGTTGTTAGCCATAAAGTGATAACCTTTAATCATTGTCTTTATTAATACAACTCACTATAAg                                                                                         <  1:69240/295‑1 (MQ=255)`  
                                                                                                                    `tgtgtataagagacaggTCTTTTGGTGTTACCTTGAGTGTCTCTCTGTGT‑‑‑CCTATCTGTTACAGTCTCCTAAAGTATCCTCCTAAAGTCACCTCCTAACGTCCATCCTAAAGCC‑AACACCTAAAGCCTACACCTAAAGACCCATCAAGTCAACGCCTATCTTAAAGTTTAAACA‑‑‑‑‑‑‑‑‑‑‑‑‑TAAAGACCAGACC‑TAAAGACCAGACCTAAAGACACTACATAAAGACCAGACCTAAAGACGCCTTGTTGTTAGCCATAAAGTGATAACCTTTAATCATTGTCTTTATTAATACAACTCACTATAAGga                                                                                       <  1:75570/285‑1 (MQ=255)`  
                                                                                                                    `gATGACCCTTGGGTTTGTCTTTGGGTGTTACCTTGAGTGTCTCTCTGTGT‑C‑CCTATCTGTAACAGTCTCCTAAAGTATCCTCCTAAAGTCACCTCCTAACGTCCATCCTAAAGCC‑AACACCTAAAGCCTACACCTAAAGACCCATCAAGTCAACGCCTATCTTAAAGTTTAAACA‑‑‑‑‑‑‑‑‑‑‑‑‑TAAAGACCAGACC‑TAAAGACCAGACCTAAAGACACTACATAAAGACCAGACCTAAAGACGCCTTGTTGTTAGCCATAAAGTGATAACCTTTAATCATTGTCTTTATTAATACAACTCACTATAAgg                                                                                        <  1:194352/301‑1 (MQ=255)`  
                                                                                                                    `gATGACCCTTGGGTGTGTCTGTGGGTGTTACCTTGAGTGTCTCTCTGTGT‑C‑CCTATCTGTAACAGTCTCCTAAAGTATCCTCCTAAAGTCACCTCCTAACGTCCATCCTAAAGCC‑AACACCTAAAGCCTACACCTAAAGACCCATCAAGTCAACGCCTATCTTAAAGTTTAAACA‑‑‑‑‑‑‑‑‑‑‑‑‑TAAAGACCAGACC‑TAAAGACCAGACCTAAAGACACTACATAAAGACCAGACCTAAAGACGCCTTGTTGTTAGCCATAAAGTGATAACCTTTAATCATTGTCTTTATTAATACAACTCACTATAAgg                                                                                        <  1:335543/301‑1 (MQ=255)`  
                                                                                                                     `aTGACCCTTGGGTTTGTCTTTGGGTGTTACCTTGAGTGTCTCTCTGTGT‑C‑CCTATCTGTAACAGTCTCCTAAAGTATCCTCCTAAAGTCACCTCCTAACGTCCATCCTAAAGCC‑AACACCTAAAGCCTACACCTAAAGACCCATCAAGTCAACGCCTATCTTAAAGTTTAAACA‑‑‑‑‑‑‑‑‑‑‑‑‑TAAAGACCAGACC‑TAAAGACCAGACCTAAAGACACTACATAAAGACCAGACCTAAAGACGCCTTGTTGTTAGCCATAAAGTGATAACCTTTAATCATTGTCTTTATTAATACAACTCACTATAAGga                                                                                       <  1:106739/301‑1 (MQ=255)`  
                                                                                                                      `tGACCCTTGGGTTTGTCTTTTGGTGTTACCTTGAGTGTCTCTCTGTGT‑C‑CCTATCTGTAACAGTCTCCTAAAGTATCCTCCTAAAGTCACCTCCTAACGTCCATCCTAAAGCC‑AACACCTAAAGCCTACACCTAAAGACCCATCAAGTCAACGCCTATCTTAAAGTTTAAACA‑‑‑‑‑‑‑‑‑‑‑‑‑TAAAGACCAGACC‑TAAAGACCAGACCTAAAGACACTACATAAAGACCAGACCTAAAGACGCCTTGTTGTTAGCCATAAAGTGATAACCTTTAATCATTGTCTTTATTAATACAACTCACTATAAGgag                                                                                      <  1:123987/301‑1 (MQ=255)`  
                                                                                                                      `cggagatgtgtataagagacaggTGTTACCTTGAGTGTCTCTCTGTGT‑C‑CCTATCTGTAACAGTCTCCTAAAGTATCCTCCTAAAGTCACCTCCTAACGTCCATCCTAAAGCC‑AACACCTAAAGCCTACACCTAAAGACCCATCAAGTCAACGCCTATCTTAAAGTTTAAACA‑‑‑‑‑‑‑‑‑‑‑‑‑TAAAGACCAGACC‑TAAAGACCAGACCTAAAGACACTACATAAAGACCAGACCTAAAGACGCCTTGTTGTTAGCAATAAAGTGATAACCTTTAATCATTGTCTTTATTAATACAACTCACTATAAGgag                                                                                      <  1:51311/280‑1 (MQ=255)`  
                                                                                                                       `gACCCTTGGGTTTGTCTTTGGGTGTTACCTTGAGTGTCTCTCTGTGT‑C‑CCTATCTGTAACAGTCTCCTAAAGTATCCTCCTAAAGTCACCTCCTAACGTCCATCCTAAAGCC‑AACACCTAAAGCCTACACCTAAAGACCCATCAAGTCAACGCCTATCTTCAAGTTTAAACA‑‑‑‑‑‑‑‑‑‑‑‑‑TAAAGACCAGACC‑TAAAGACCAGACCTAAAGACACTACATAAAGACCAGACCTAAAGACGCCTTGTTGTTAGCCATAAAGTGATAACCTTTAATCATTGTCTTTATTAATACAACTAACTATAAGgaga                                                                                     >  1:66078/1‑301 (MQ=255)`  
                                                                                                                       `cgcccTTGGGTTTTTCTTTGGGTGTTACCTTGAGTGTCTCTCTGTGTCC‑CCTATCTGTTACAGTCTCCTAAAGTATCCTCCTAAAGTCACCTCCTAACGTCCATCCTAAAGCC‑AACACCTAAAGCCTACACCTAAAGACCCATCAAGTCAACGCCTATCTTAAAGTTTAAACA‑‑‑‑‑‑‑‑‑‑‑‑‑TAAAGACCAGACC‑TAAAGACCAGACCTAAAGACACTACATAAAGACCAGACCTAAAGACGCCTTGTTGTTAGCCATAAAGTGATAACCTTTAATCATTGTCTTTATTAATACAACTCACTATAAGgag                                                                                      <  1:90563/299‑1 (MQ=255)`  
                                                                                                                         `cccTTTGGTTTGTCTTTGGGTGTTACCTTGAGTGTCTCTCTGTGT‑C‑CCTATCTG‑TACAGTCTCCTAAAGTATCCTCCTAAAGTCACCTCCTAACGTCCATCCTAAAGCC‑AACACCTAAAGCCTACACCTAAAGACCCATCAAGTCAACGCCTATCTTAAAGTTTAAACA‑‑‑‑‑‑‑‑‑‑‑‑‑TAAAGACCAGACC‑TAAAGACCAGACCTAAAGACACTACATAAAGACAAGACCTAAAGACGCCTTGTTGTTAGCCATAAAGTGATAACCTTTAATCATTGTCTTTATTAATACAACTCACTATACGGAGAGAc                                                                                  >  1:45939/1‑301 (MQ=255)`  
                                                                                                                         `cccTTGTGTTTGTCTTTGGGTGTTACCTTGAGTGTCTCTCTGTGT‑C‑CCTATCTGTTACAGTCTCCTAAAGTCACCTCCTAAAGTCACCTCCTAACGTCCATCCTAAAGCC‑AACACCTAAAGCCTACACCTAAAGACCCATCAAGTCAACGCCTATCTTAAAGTTTAAACA‑‑‑‑‑‑‑‑‑‑‑‑‑TAAATACCAGACC‑TAAAGACCAGACCTAAAGACACTACATAAAGACCAGACCTAAAGACGCCTTGTTGTTAGCCATAAAGTGATAACCTTTAATCATTGTCTTTATTAATACAACTCACTATAAGgagaga                                                                                   <  1:21155/301‑1 (MQ=255)`  
                                                                                                                           `cTTGGGTTTGTCTTTGGGTGTTACCTTGAGTGTCTCTCTGTGT‑C‑CCTATCTGTAACAGTCTCCTAAAGTATCCTCCTAAAGTCACCTCCTAACGTCCATCCTAAAGCC‑AACACCTAAAGCCTACACCTAAAGACCCATCAAGTCAACGCCTATCTTAAAGTTTAAACA‑‑‑‑‑‑‑‑‑‑‑‑‑TAAAGACCAGACC‑TAAAGACCATACCTAAAGACACTACATAAAGACCAGACCTAAAGACGCCTTGTTGTTAGCCATAAAGTGATAACCTTTAATCATTGTCTTTATTAATACAACTCACTATAAGgactgtct                                                                                 >  1:250786/1‑295 (MQ=255)`  
                                                                                                                               `ggTTTGTCTTTGGGTGTTACCTTGAGTGTCTCTCTGTGT‑C‑CCTATCTGTAACAGTCTCCTAAAGTATCCTCCTAAAGTCACCTCCTAACGTCCATCCTAAAGCC‑AACACCTAAAGCCTACACCTAAAGACCCATCAAGTCAACGCCTATCTTAAAGTTTAAACA‑‑‑‑‑‑‑‑‑‑‑‑‑TAAAGACCAGACC‑TAAAGACCAGACCTAAAGACACTACATAAAGACCAGACCTAAAGACGCCTTGTTGTTAGCCATAAAGTGATAACCTTTAATCATTGTCTTTATTAATACAACTCACTATAAGGAGAGACAACtt                                                                             >  1:224233/1‑301 (MQ=255)`  
                                                                                                                                   `tGTCTTTGGGTGTTACCTTGAGTGTCTCTCTGTGT‑C‑CCTATCTGTAACAGTCTCCTAAAGTATCCTCCTAAAGTCACCTCCTAACGTCCATCCTAAAGCC‑AACACCTAAAGCCTACACCTAAAGACCCATCAAGTCAACGCCTATCTTAAAGTTTAAACA‑‑‑‑‑‑‑‑‑‑‑‑‑TAAAGACCAGACC‑TAAAGACCAGACCTAAAGACACTACATAAAGACCAGACCTAAAGACGCCTTGTTGTTAGCCATAAAGTGATAACCTTTAATCATTGTCTTTATTAATACAACTCACTATAAGGATAGACAACTTAAag                                                                         <  1:240634/301‑1 (MQ=255)`  
                                                                                                                                    `gTCTTTGGGTGTTACCTTGAGTTTCTCTCTGTGT‑C‑CCTATCTGTAACAGTCTCCTAAAGTATCCTCTTAAAGTCACCTCCTAACGTCCATCCTAAAGCC‑AACACCTAAAGCATACACCTAAAGACCCATCAAGTCAACGCCTATCTTCAAGTTTAAACA‑‑‑‑‑‑‑‑‑‑‑‑‑TAAAGACCAGACA‑TACAGACCAGACCTAAAGACACTATATAAAGAACAGACCTAAAGACGCCTTGTTGTTAGCCATCAAGTGATACCATTTAATAATTGTCTTAATTACACAAACTCAATATTAGGAGCGCCAACTTacaaa                                                                        >  1:324709/1‑297 (MQ=255)`  
                                                                                                                                    `gTCTTTGGGTGTTACCTTGAGTGTCTCTCTGTGT‑C‑CCTATCTGTAACAGTCTCCTAAAGTATCCTCCTAAAGTCACCTCCTAACGTCCATCCTAAAGCC‑AACACCTAAAGCCTACACCTAAAGACCCATCAAGTCAACGCCTATCTTAAAGTTTAAACA‑‑‑‑‑‑‑‑‑‑‑‑‑TAAAGACCAGACC‑TAAAGACCAGACCTAAAGACACTACATAAAGACCAGACCTAAAGACTCCTTGTTGTTAGCCATAAAGTAATAACCTTTAATCATTGTCTTTATTAATACAACTCACTATAAggctgtctcttatacaca                                                                        >  1:214600/1‑285 (MQ=255)`  
                                                                                                                                    `aaagagacaggtTACCTTGAGGTGCTCTCTGTGT‑C‑CCTATCTGTAACAGTCTCCTAAAGTATCCTCCTAAAGTCACCTCCTAACGTCCATCCTAAAGCC‑AACACCTAAAGCCTACACCTAAAGAACCAACAAGTCAACGCCTATCTTAAAGTTTAAACA‑‑‑‑‑‑‑‑‑‑‑‑‑TAAAGACCAGACC‑TAAAGACCAGACCTAAAGACACTACATAAAGACCAGACCTAAAGACGCCTTGTTGTTAGCCATAAAGTGATAACCTTTAATCATTGTCTTTATTAATACAACTCACTATAAGGAGAGACAACTTAAaga                                                                        <  1:95145/291‑1 (MQ=255)`  
                                                                                                                                     `aacaggggTGTTACCTTGAGTGTCTCTCTGTGTCC‑CCTATCTGTAACAGTCTCCTAAAGTAACCTCCTAAAGTCACCTCCTAACGTCCATCCTAAAGCC‑AACACCTAAAGACTACACCTAAAGACCCATCAAGCCAACGCCTATCTTAAAGTTTAAACA‑‑‑‑‑‑‑‑‑‑‑‑‑TAAAGACCAGACC‑TAAAGACCAGACCTAAAGACACTACATAAATACCAGACCTAAAGACGCCTTGTTGTTAGCCATAAAGTGATAACCTTTAATCATTGTCTTTATTAATACAACTCACTATAAGGAGAGACAACTTAAaga                                                                        <  1:234232/296‑1 (MQ=255)`  
                                                                                                                                            `gtgtTACCTTGAGTGTCTCTCTGTGT‑C‑CCTATCTGTAACAGTCTCCTAAAGTATCCTCCTAAAGTCACCTCCTAACGTCCATCCTAAAGCC‑AACACCTAAAGCCTACACCTAAAGACCCATCAAGTCAACGCCTATCTTAAAGTTTAAACA‑‑‑‑‑‑‑‑‑‑‑‑‑TAAAGACCAGACC‑TAAAGACCAGACCTAAAGAAACTACATAAAGACCAGACCTAAAGACGCCTTGTTGTTAGCCATAAAGTGATAACCTTTAATCATTGTCTTTATTAATACAACTCACTATAAGGAGAGACAACTTAAAGAGACTTaaa                                                                >  1:37845/1‑301 (MQ=255)`  
                                                                                                                                              `tttACCTTGAGTGTCTCTCTGTGT‑C‑CCTATCTGTAACAGACTCCTAAAGTATCCTCCTAAAGTCACCTCCTAACGTCCATCCTAAAGCC‑AACACCTAAAGCCTACACCTAAAGACCCATCAAGTCAACGCCTATCTTAAAGTTTAAACA‑‑‑‑‑‑‑‑‑‑‑‑‑TAAAGACCAGACC‑TAAAGACCAGACCTATAGACACTACATAAAGACCAGACCTAAAGACGCCTTGTTGTTAGCCATAAAGTGATAACCTTTAATCATTGTCTTTATTAATACAACTCACTATAAGGAGAGACAACGTAAAGAGACTTAAAAg                                                              <  1:231570/300‑1 (MQ=255)`  
                                                                                                                                               `ttACCTTGAGTGTCTCTCTGTGTCC‑CCTTGCTGTTACAGTCTCCTAAAGTATCCTCCAAAAGTCACCTCCTAACTTCCATCCTAGAGCC‑AACACCTAAAGCCTACACCTAAAGACCCATCAAGTCAACGCCTATCTTAAAGTTTAGACA‑‑‑‑‑‑‑‑‑‑‑‑‑TAAAGACCAGACC‑TAAAGACCAGACCTAAAGACACTACATAAAGACCAGACCTAAAGACGCCTTGTTGTTAGCCATAAAGTGATAACCTTTAATCATTGTCTTTATTAATACAACTCACTATAAGGAGAGACAACTTAAAGAGACTTAAAAg                                                              <  1:292579/301‑1 (MQ=255)`  
                                                                                                                                               `gagacaggAGTGTCTCTCTGTGT‑C‑CC‑ATCTGTTACAGTCTCCTAAAGTATCCTCCTAAAGTCACCTCCTAACGTCCATCCTAAAGCC‑AACACCTAAAGCCTACACCTAAAGACCCATCAAGTCAACGCCTATCTTAAAGTTTAAACATAAAGACCAGACCTAAAGACCAGACC‑TAAAGACCAGACCTAAAGACACTACATAAAGACCAGACCTAAAGACGCCTTGTTGTTAGCCATAAAGTGATAACCTTTAATCATTGTCTTTATTAATACAACTCACTATAAGGAGAGACAACTTAAag                                                                         <  1:218064/294‑1 (MQ=255)`  
                                                                                                                                                `tACCTTGAGTGTCTCTCTGTGT‑C‑CCTATCTGTAACAGTCTCCTAAAGTATCCTCCTAAAGTCACCTCCTAACGTCCATCCTAAAGCC‑AACACCTAAAGCCTACACCTAAAGACCCATCAAGTCAACGCCTATCTTAAGGTTTAAACA‑‑‑‑‑‑‑‑‑‑‑‑‑TAAAGACCAGACC‑TAAAGACCAGACCTAAAGACACTACATCAAGACCAGACATACAGACGCCTTGTTGTTAGACATAAAGTGATAACCTTTAATCATTGTCTTTATTAATACAACTCACTATAAGGAGAGACAACTTAAAGAGACTTACAAGAt                                                            >  1:137692/1‑301 (MQ=255)`  
                                                                                                                                                `ggggctcggagatgtgtataagagacagaTCTGTAACAGTCTCCTAAAGGATCCTCCTAAAGTCACCTCCTAACGTCCATCCTAAAGCC‑AACACCTAAAGCCTACACCTAAAGACCCATCAAGTCAACGCCTATCTTAAAGTTTAAACA‑‑‑‑‑‑‑‑‑‑‑‑‑TAAAGACCAGACC‑TAAAGACCAGACCTAAAGACACTACATAAAGACCAGACCTAAAGACGCCTTGTTGTTAGCCATAAAGTGA‑AACCTTTAATCATTGTCTTTATTAATACAACTCACTATAAGGAGAGACAACTTAAAGAGACTTAAAAGa                                                             <  1:284733/273‑1 (MQ=255)`  
                                                                                                                                                        `gtgtCTCTCTGTGT‑C‑CCTATCTGTAACAGTCTCCTAAAGTATCCTCCTAAAGTCACCTCCTAACGTCCATCCTAAAGCC‑AACACCTAAAGCCTACACCTAAAGACTCATCAAGTCAACGCCTATCTTAAAGTTTAAACA‑‑‑‑‑‑‑‑‑‑‑‑‑TAAAGACCAGACC‑TAAAGACCAGACCTAAAGACACTACATAAAGACCAGACCTAAAGACGCCTTGTTGTTAGCCATAAAGGTCTCACCTTTAATCATTGTCTTTATTAATACAACTCACTATAAGGAGAGACAACTTAAAGAGACTTACAAgctgtctctta                                                    >  1:141055/1‑291 (MQ=255)`  
                                                                                                                                                        `gtgtCTCTCTGTGT‑C‑CCTATCTG‑TACAGTCTCCTAAAGTATCCTCCTAAAGTCACCTCCTAACGTCCATCCTAAAGCC‑AACACCTAAAGCCTACACCTAAAGACCCATCAAGTCAACGCCTATCTTAAAGTTTAAACA‑‑‑‑‑‑‑‑‑‑‑‑‑TAAAGACCAGACA‑TAAAGACCAGACCTAAAGACACTACATAAAGACCAGACCTAAAGACGCCTTGTTGTTAGCCATAAAGTGATAACCTTTAATCATTGTCTTTATTAATACAACTCACTATAAGGAGATACAACTTAAAGAGACTTAAAAGATTAATTTaaa                                                   >  1:148247/1‑301 (MQ=255)`  
                                                                                                                                                            `ctctctGTGTCC‑CCTATCTGTTACAGTCTCCTAAAGTATCCTCCTAAAGTCACCTCCTAACGTCCATCCTAAAGCC‑AACACCTAAAGCCTACACCTAAAGACCCATCAAGTCAACGCCTATCTTAAAGTTTAAACA‑‑‑‑‑‑‑‑‑‑‑‑‑TAAAGACCAGACC‑TAAAGACCAGACCTAAAGACACTACATAAAGACCAGACCTAAAGACGCCTTGTTGTTAGCCATAAAGTGATAACCTTTAATCATTGTCTTTATTAATACAACTCACTATAAGGAGAGACAACTTAAAGAGACTTAAAAGATTAATTTAAAAt                                                 <  1:306825/301‑1 (MQ=255)`  
                                                                                                                                                               `tctGTGTCC‑CCTATCTGTTACAGTCTCCTAAAGTATCCTCCTAAAGTCACCTCCTAACGTCCATCCTAAAGCC‑AACACCTAAAGCCTACACCTAAAGACCCATCAAGTCAACGCCTATCTTAAAGTTTAAACA‑‑‑‑‑‑‑‑‑‑‑‑‑TAAAGACCAGACC‑TAAAGACCAGACCTAAAGACACTACATAAAGACCAGACCTAAAGACGCCTTGTTGTTAGCCATAAAGTGATAACCTTTAATCATTGTCTTTATTAATACAACTCACTATAAGGAGAGACAACTTAAAGAGACTTAAAAGATTAATTTAAAATTTa                                              >  1:178475/1‑301 (MQ=255)`  
                                                                                                                                                                       `c‑ccTATCTGTTACAGTCTCCTAAAGTATCCTCCTAAAGTCACCTCCTAACGTCCATCCTAAAGCC‑AACACCTAAAGCCTACACCTAAAGATCCATCAAGTCAACGCCTATCTTAAAGTTTAAACA‑‑‑‑‑‑‑‑‑‑‑‑‑TAAAGACCAGACC‑TAAAGACCAGACCTAAAGACACTACATAAAGACCAGACCTAAAGACGCCTTGTTGTTAGCCATAAAGTGATAACCTTTAATCATTGTCTTTATTAATACAACTCACTATAAGGAGAGACAACTTAAAGAGACTTAAAAGATTAATTTAAAATTTATCaaaaaa                                      >  1:110256/1‑300 (MQ=255)`  
                                                                                                                                                                       `c‑ccTATCTG‑TACAGTCTCCTAAAGTATCCTCCTAAAGTCACCTCCTAACGTCCATCCTAAAGCC‑AACACCTAAAGCCTACACCTAAAGACCCATCAAGTCAACGCCTATCTTAAAGTTTAAACA‑‑‑‑‑‑‑‑‑‑‑‑‑TAAAGACCAGACC‑TAAAGACCAGACCTAAAGACACTACATAAAGACCAGACCTAAAGACGCCTTGTTGTTAGCCATAAAGTGATAACCTTTAATCATTGTCTTTATTAATACAACTCACTATAAGGAGAGACAACTTAAAGAGACTTAAAAGATTAATTTAAAATTTATCAAAAcga                                     >  1:188082/1‑301 (MQ=255)`  
                                                                                                                                                                              `cTGTTACAGTCTCCTAAAGTATCCTCCTAAAGTCACCTCCTAACGTCCATCCTAAAGCC‑AACACCTAAAGCCTACACCTAAAGACCCATCAAGTCAACGCCTATCTTAAAGTTTAAACA‑‑‑‑‑‑‑‑‑‑‑‑‑TAAAGACCAGACC‑TAAAGACCAGACCTAAAGACACTACATAAAGACCAGACCTAAAGACGCCTTGTTGTTAGCCATAAAGTGATAACCTTTAATCATTGTCTTTATTAATACAACTCACTATAAGGAGAGACAACTTAAAGAGACTTAAAAGATTAATTTAAAATTTATCAAAAAGAGTAtt                                >  1:282428/1‑301 (MQ=255)`  
                                                                                                                                                                                 `gtACAGTCTCCTAAAGTATCCTCCTAAAGTCACCTCCTAACGTCCATCCTAAAGCC‑AACACCTAAAGCCTACACCTAAAGACCCATCAAGTCAACGCCTATCTTAAAGTTTAAACA‑‑‑‑‑‑‑‑‑‑‑‑‑TAAAGACCAGACC‑TAAAGACCAGACCTAAAGACACTACATAAAGACCAGACCTAAAGACGCCTTGTTGTTAGCCATAAAGTGATAACCTTTAATCATTGTCTTTATTAATACAACTCACTATAAGGAGAGACAACTTAAAGAGACTTAAAAGATTAATTTAAAATTTATCAAAAAGAGTATTGAc                             <  1:79162/300‑1 (MQ=255)`  

                                                                                                                                                                                  `|`                                                                                                                                                                                                                                                                                                                                                       
`‑‑‑‑‑‑‑‑‑TCTCACAGTGTACGGACCTAAAGTT‑CCCCCATAGGGGGTACCTAAAGCCCAGCCAATCACCTAAAGTCAACCTTCGGTTGACCTTGAGGGTTCCCTAAGGGTTGGGGATGACCCTTGGGTTTGTCTTTGGGTGTTACCTTGAGTGTCTCTCTGTGT‑C‑CCTATCTGTTACAGTCTCCTAAAGTATCCTCCTAAAGTCACCTCCTAACGTCCATCCTAAAGCC‑AACACCTAAAGCCTACACCTAAAGACCCATCAAGTCAACGCCTATCTTAAAGTTTAAACA‑‑‑‑‑‑‑‑‑‑‑‑‑TAAAGACCAGACC‑TAAAGACCAGACCTAAAGACACTACATAAAGACCAGACCTAAAGACGCCTTGTTGTTAGCCATAAAGTGATAACCTTTAATCATTGTCTTTATTAATACAACTCACTATAAGGAGAGACAACTTAAAGAGACTTAAAAGATTAATTTAAAATTTATCAAAAAGAGTATTGACTTAAAGTCTAACCTATAGGATACTTAC  >  NC_001604.1/1‑493`

  

Alignment Legend

Aligned base mismatch/match (shaded by quality score): `ATCG/ATCG` `< 3 ≤` `ATCG/ATCG` `< 11 ≤` `ATCG/ATCG` `< 23 ≤` `ATCG/ATCG` `< 34 ≤` `ATCG/ATCG` `< 38 ≤` `ATCG/ATCG`

Unaligned base: `atcg`    Masked matching base: `atcg`    Alignment gap: `‑`    Deleted base: `‑`
